# Combined NY1301 and DDMP on Sleep and Autonomic Function in Summer: A Randomized Clinical Trial

**DOI:** 10.3390/jcm15114175

**Published:** 2026-05-28

**Authors:** Masato Tomizawa, Takuya Sugimoto, Masanori Fukao

**Affiliations:** 1 Development Laboratories, Nissin York Co., Ltd., 272 Kamimurakimi, Hanyu, Saitama 348-8549, Japan; masanori.fukao@nissin.com; 2 Global Innovation Center, Nissin Foods Holdings Co., Ltd., 2100 Tobukimachi, Hachioji-shi, Tokyo 192-0001, Japan; takuya.sugimoto@nissin.com

**Keywords:** *Lacticaseibacillus paracasei* subsp. *paracasei* NY1301, 2,3-dihydro-3,5-dihydroxy-6-methyl-4H-pyran-4-one, summer heat fatigue, sleep quality, randomized double-blind parallel-group trial, autonomic nervous system function

## Abstract

**Objectives:** Summer heat can impair sleep and contribute to autonomic dysregulation and fatigue-related symptoms. Because heat exposure may induce oxidative stress and gut-related dysfunction, we hypothesized that combined intake of the probiotic *Lacticaseibacillus paracasei* subsp. *paracasei* NY1301 and the antioxidant 2,3-dihydro-3,5-dihydroxy-6-methyl-4H-pyran-4-one (DDMP) would improve sleep, fatigue, and autonomic recovery during the summer. **Methods:** In this randomized, double-blind, placebo-controlled, parallel-group trial, 171 healthy adults were administered a beverage containing NY1301 and DDMP or placebo once daily for 8 weeks during the summer. The primary outcome was subjective fatigue assessed by a web-based daily log. Secondary outcomes included visual analog scale (VAS) fatigue scores, weekly questionnaire items, sleep parameters, and heart rate variability before and after mental arithmetic tasks. **Results:** No statistically significant between-group difference was observed in the primary outcome, subjective fatigue assessed using the web-based daily log. However, among secondary outcomes, sleep onset latency at week 8 was significantly shorter in the NY1301 + DDMP group (mean difference: −2.07 min, 95% CI −3.96 to −0.17; *p* = 0.033), and pre-task fatigue VAS at week 8 was lower (mean difference: −6.79, 95% CI −13.6 to −0.3; *p* = 0.047). At week 8, the NY1301 + DDMP group showed a greater increase in LF/HF during the task (mean difference: 1.32, 95% CI 0.06 to 2.58; *p* = 0.040), greater decrease in LF/HF during recovery (mean difference: −1.34, 95% CI −2.39 to −0.30; *p* = 0.012), and greater increase in HF during recovery (mean difference: 140, 95% CI 43 to 239; *p* = 0.005). In an exploratory analysis, the temperature-associated rise in nocturnal respiratory rate was significantly attenuated in the NY1301 + DDMP group (group × temperature interaction, *p* < 0.05). **Conclusions:** The primary outcome, subjective fatigue assessed using the web-based daily log, was not significantly different between groups during the summer study period. However, combined intake of NY1301 and DDMP was associated with shorter sleep onset latency, lower pre-task fatigue under standardized conditions, attenuation of the temperature-associated rise in nocturnal respiratory rate, and more pronounced post-stress autonomic recovery during the summer. These findings suggest a potential physiological benefit of this combined nutritional approach under summer conditions and warrant confirmation in independent trials. The study was funded by Nissin York Co., Ltd., and potential conflicts of interest related to sponsor-affiliated authors are disclosed in the manuscript.

## 1. Introduction

Global warming has increased surface temperatures worldwide [1], leading to a marked increase in summer heat severity. Severe environmental conditions during the summer months frequently lead to summer heat fatigue, characterized by a constellation of nonspecific symptoms, such as persistent malaise, loss of appetite, and diminished sleep quality [2]. Furthermore, an individual’s sleep status is a critical determinant of resilience to heat stress; those with poor sleep patterns experience increased fatigue as ambient temperatures rise [3]. In Japan, this condition is commonly understood as summer heat fatigue (*natsubate*)—a summer-associated state of nonspecific complaints rather than an acute heat illness such as heatstroke, characterized by persistent malaise or fatigue, sleep disturbance, loss of appetite, poor circulation, and other nonspecific complaints [4,5,6].

The accumulation of summer heat fatigue has significant socioeconomic impacts, specifically through impairing labor productivity. A recent report has indicated that approximately 60% of workers exposed to elevated temperatures suffer from significant productivity loss, a decline that becomes particularly pronounced when ambient temperatures exceed 35 °C [7]. It has been hypothesized that disruption of the autonomic nervous system is involved in the underlying pathophysiology of these symptoms [5].

Heat stress induces oxidative stress and impairs intestinal function [8,9,10]. Oxidative stress promotes sympathetic activation; in experimental models, reducing central reactive oxygen species levels attenuates sympathetic nerve activity [11]. In addition, impaired intestinal barrier function may facilitate endotoxin translocation and increase circulating pro-inflammatory cytokines [10]. Notably, inflammatory signals, such as cytokines, activate central autonomic pathways, resulting in increased sympathetic activity [10,12]. Therefore, heat-induced oxidative stress and gut-derived inflammatory signaling may contribute to sympathetic hyperactivation, serving as important pathophysiological drivers of summer heat fatigue.

Given the potential roles of oxidative stress and gut dysbiosis in heat-induced sympathetic hyperactivation, nutritional approaches targeting these pathways may represent a practical strategy to mitigate summer heat fatigue. Clinical studies have reported that the intake of antioxidant-related food components is associated with reduced subjective fatigue in healthy adults [13,14]. Moreover, in a randomized, double-blind, placebo-controlled trial, intake of a lactic acid bacteria (LAB)-fermented dairy product reduced fatigue symptoms in healthy individuals experiencing summer heat fatigue [4]. Clinical reports also support the plausibility of combining probiotic-related and antioxidant-related interventions. For example, a probiotic supplement combined with an antioxidant-rich elderberry extract improved well-being-related outcomes, while a fermented plant extract produced using lactic acid bacteria and bifidobacteria improved subjective fatigue in healthy adults [15,16]. These findings suggest that combining an antioxidant component with a probiotic represents a rational strategy to address summer heat fatigue by simultaneously attenuating heat-induced oxidative stress and improving gut-related homeostasis.

2,3-Dihydro-3,5-dihydroxy-6-methyl-4H-pyran-4-one (DDMP) is an antioxidant compound formed during the Maillard reaction. It contributes to the antioxidative activity of beverages fermented by LAB [17]. In randomized, double-blind, placebo-controlled trials, intake of DDMP-containing LAB beverages has been reported to improve cognitive function [18], skin condition [19], and physical performance [20]. *Lacticaseibacillus paracasei* subsp. *paracasei* NY1301, used in fermented beverages, is a probiotic strain with evidence for improving bowel function [21], and a beverage containing NY1301 has also been reported to improve subjective fatigue and sleepiness on waking in adults with fatigue [22]. Because DDMP can be generated during the manufacturing process of LAB beverages and both components have been consumed as foods, no specific safety concerns are anticipated for their combined intake. DDMP targets oxidative stress-related pathways, whereas NY1301 may support gut-related homeostasis. Thus, their combined intake may represent a dual-pathway nutritional strategy for summer-related autonomic dysregulation. However, their combined efficacy for summer heat fatigue-related symptoms has not been evaluated.

We conducted a randomized, double-blind, placebo-controlled, parallel-group trial of the effects of the combined intake of the probiotic NY1301 and antioxidant DDMP on sleep-related outcomes, fatigue-related symptoms under standardized conditions and autonomic recovery from psychological stress in healthy adults during the summer.

## 2. Materials and Methods

### 2.1. Study Procedures

Our study protocol was approved by the Shiba Palace Clinic Ethics Review Committee (Tokyo, Japan), in accordance with the Declaration of Helsinki and the Ethical Guidelines for Life Sciences and Medical Research Involving Human Subjects. This study was registered with the University Hospital Medical Information Network Clinical Trials Registry as UMIN000057607 on 15 April 2025, and it was conducted in compliance with the protocol. Written informed consent was obtained from all participants. A protocol amendment to the statistical analysis plan was prepared during the study and approved by the Shiba Palace Clinic Ethics Review Committee. The amendment specified that, in addition to the prespecified analyses, exploratory analyses of changes in autonomic nervous system indices (LF/HF ratio and HF component) from pre-task to immediately post-task and during recovery would be performed to inform the mechanistic interpretation of the trial. These autonomic-related analyses were finalized after database lock and unblinding; however, because the dataset had already been locked, no modification of the underlying data was possible. These analyses were exploratory in nature and not intended as confirmatory tests of efficacy; they were performed to deepen the mechanistic interpretation of the trial rather than to evaluate the primary or other prespecified efficacy outcomes. The post-lock timing of these exploratory mechanistic analyses was disclosed to the Editor at the time of initial submission. All analyses reported in this manuscript were performed using the locked dataset. This study was conducted by a contract research organization, EUPHORIA Co., Ltd. (Tokyo, Japan), from April 2025 to December 2025. Recruitment and intervention were conducted from May 2025 to September 2025 at EUPHORIA Co., Ltd.

### 2.2. Participants

We recruited healthy Japanese adults aged 20 to 59 years who had previously experienced summer heat fatigue. Participants were confirmed to be in good health based on pre-study screening assessments. All participants received a thorough explanation of the study, fully understood its contents, and voluntarily provided written informed consent.

The exclusion criteria were as follows: (1) engaging in regular exercise (defined as exercising at least twice a week for 30 min or more per session), (2) BMI < 18.5 kg/m^2^ or ≥30.0 kg/m^2^, (3) having a disease, undergoing treatment, or having a history of serious diseases such as diabetes, liver disease, kidney disease, heart disease, or cardiovascular disorders, (4) current illness requiring treatment or a history of serious illness requiring pharmacological treatment, (5) consuming fermented milk or LAB beverages at least twice a week during the 3 months prior to providing informed consent, (6) regular use of over-the-counter medicines, quasi-drugs, or pharmacist-guided medicines for nutritional supplementation or anti-fatigue purposes during the 3 months prior to providing informed consent, (7) allergies to dairy products or lactose intolerance, (8) participation in another clinical study within 1 month before informed consent or planned participation in another clinical study after enrollment, (9) likely to change lifestyle habits, including diet and exercise, during the study period, (10) deemed unsuitable for participation by the study physician based on questionnaire responses, (11) planning to travel abroad, donate blood, become pregnant, or breastfeed during the study period, and (12) judged by the clinical investigator to be unsuitable for participation.

The sample size was determined with reference to previous randomized, double-blind, placebo-controlled, parallel-group trials evaluating the effects of NY1301 and DDMP involving approximately 160 participants [20,22].

### 2.3. Study Design

**Figure 1:**
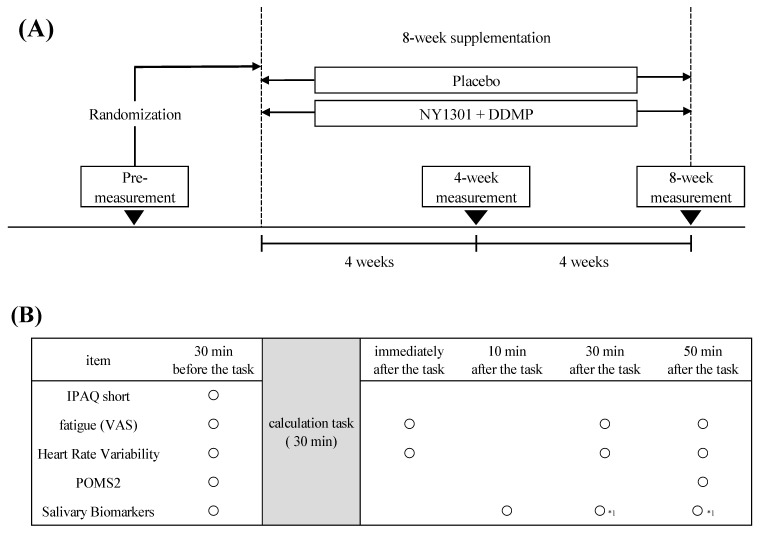
Study design: (**A**) NY1301, *Lacticaseibacillus paracasei* subsp. *paracasei* NY1301; DDMP, 2,3-dihydro-3,5-dihydroxy-6-methyl-4H-pyran-4-one. (**B**) Examination schedule at the clinic visit. VAS: Visual Analog Scale.

This study was designed as a randomized, double-blind, placebo-controlled study. Participants consumed the test foods for 8 weeks between July and September 2025. A schematic of the study is shown in Figure 1.

Participants were assessed at three time points: before the intake of the test foods (baseline), after 4 weeks of intake (week 4), and after 8 weeks of intake (week 8). Participants were randomized after the baseline assessment. During the 8-week intervention period, participants were required to record their daily compliance and physical condition using a web-based diary. In addition, participants wore an Oura Ring (Oura Health Oy, Oulu, Finland) throughout the study period to objectively monitor sleep quality.

At each clinic visit, a 30 min mental arithmetic task was conducted as a psychological stressor, adapted from a previously described method [23]. This task required participants to perform arithmetic operations using two- to three-digit numbers. To evaluate the psychological and physiological responses to this stress challenge as well as the subsequent recovery process, multiple parameters were assessed: subjective fatigue (visual analog scale; VAS), autonomic nervous system function, Profile of Mood States 2nd Edition (POMS2) [24,25], and salivary biomarkers (α-amylase and cortisol). The specific items measured and their respective timing—ranging from 30 min before task initiation to 50 min after task completion—are illustrated in the experimental timeline in Figure 1. In addition, physical activity levels were evaluated at the beginning of each visit using the International Physical Activity Questionnaire (IPAQ) short form [26,27].

Stratified randomization was performed according to sex and age. Sex was stratified as male or female, and age was stratified into two categories based on the median age. The allocation sequence was generated by the study controller. Participant management, data collection, and study operations were conducted by an external contract research organization, EUPHORIA Co., Ltd. Treatment allocation codes were assigned by an independent administrator and kept sealed until the database was locked. All participants, investigators, and study personnel remained blinded to treatment allocation throughout the study period. In addition, exclusions from the efficacy analysis population were finalized before unblinding, and adverse events were assessed by the clinical investigator.

### 2.4. Test Foods

Participants were randomly assigned to either the intervention or placebo group. The intervention group was instructed to consume one bottle (65 mL) of a LAB beverage containing 6.0 × 10^10^ colony-forming units (CFU) of *Lacticaseibacillus paracasei* subsp. *paracasei* NY1301 and 2.2 mg of DDMP once daily for 8 weeks. The placebo group consumed an identical volume of a beverage that contained neither NY1301 nor DDMP. Both beverages were produced by Nissin York Co., Ltd. (Saitama, Japan) and were indistinguishable in color, flavor, and appearance.

### 2.5. Outcome Measures

The primary outcome was subjective fatigue assessed using a web-based daily log. Secondary outcomes were subjective fatigue assessed using VAS, sleep parameters measured using a wearable device, symptoms of summer heat fatigue assessed using the web-based daily log, cognitive task performance, autonomic nervous system function, salivary biomarkers, POMS2 scores, and IPAQ short-form scores.

Safety was evaluated based on adverse events recorded throughout the study period and assessed by the clinical investigator.

### 2.6. Web-Based Daily Log

At baseline (week 0) and throughout the 8-week intervention period, participants recorded compliance and subjective conditions using a web-based daily log. Compliance with the study products was recorded daily. Concomitant use of medicines, quasi-drugs, and dietary supplements was recorded daily. Occurrence of physical discomfort or illness was also recorded daily.

In addition, participants completed a web-based questionnaire consisting of 12 items related to summer heat fatigue (dizziness/unsteadiness, tinnitus, stiff shoulders, lower back pain, appetite, fatigue, irritability, sensitivity to cold, general fatigue, motivation, mental clarity, and satisfaction with sleep) once weekly. Each item was rated on a 10-point scale anchored from 1 (“not at all”) to 10 (“very much”).

### 2.7. Questionnaire

At each clinic visit, participants completed questionnaires assessing subjective fatigue, POMS2, and IPAQ short form. Subjective fatigue was assessed using VAS. Participants marked their current fatigue on a 100 mm horizontal line, with the left end (0) defined as “best condition: no fatigue at all” and the right end (100) defined as “worst condition: extremely exhausted and unable to do anything.” The VAS score was calculated as the distance (mm) from the left end to the participant’s mark.

### 2.8. Autonomic Nervous System Function

Autonomic nervous system function was assessed using heart rate variability (HRV). RR intervals were recorded using a chest-strap heart rate sensor (Polar H10; Polar Electro, Kempele, Finland) and an HRV measurement application (Elite HRV; Elite HRV, Asheville, NC, USA). Measurements were performed in a seated position under spontaneous breathing for 3 min. The following HRV indices were derived: heart rate (HR), root mean square of successive differences (RMSSD), standard deviation of normal-to-normal intervals (SDNN), high-frequency power (HF), low-frequency power (LF), and the LF/HF ratio.

### 2.9. Stress Biomarkers

Two salivary biomarkers were assessed: the salivary α-amylase activity and salivary cortisol concentration.

Salivary α-amylase activity was measured using a portable salivary amylase monitor (NIPRO, Osaka, Japan). A dedicated disposable test strip was placed under the tongue for approximately 30 s and then analyzed according to the manufacturer’s instructions.

For salivary cortisol, 2 mL of saliva was collected using a funnel-type saliva collection kit. The cortisol concentration was analyzed by LSI Medience Corporation (Tokyo, Japan) using validated laboratory procedures.

### 2.10. Statistical Analysis

Baseline characteristic data are expressed as means ± SEM. Analyses were conducted using the per-protocol set (*n* = 161), which was prespecified in the statistical analysis plan as the primary population for efficacy evaluation. The per-protocol set included participants from the full analysis set (FAS, *n* = 164) who had no major protocol deviations or insufficient adherence that could materially affect the interpretation of efficacy. All exclusions from the efficacy analysis population were based on predefined criteria and were finalized prior to unblinding.

All statistical analyses were performed using R (ver. 4.5.1). Statistical significance was set at *p* < 0.05 for all analyses. A single prespecified primary outcome was defined for this trial. Secondary outcomes were analyzed to characterize related domains. No multiplicity adjustment was applied to the analyses of multiple secondary outcomes, because these analyses were intended to be supportive and exploratory. Normality was assessed using the Shapiro–Wilk test. For parameters following a normal distribution, Welch’s *t*-test was employed for intergroup comparisons. For parameters that did not follow a normal distribution, the Mann–Whitney U test was used. For sleep quality measured using a wearable device, linear mixed-effects model analyses were performed with time course and test-food contents, baseline values for each measurement item, fixed effects for the interaction between time course and test-food contents, and participant ID was included as a random effect. For these linear mixed-effects models, statistical comparisons were conducted based on the estimated marginal means. As an additional exploratory analysis, we examined the association between weekly average ambient temperature and respiratory rate during sleep using a linear mixed-effects model. The dependent variable was respiratory rate during sleep, measured by the wearable device. Fixed effects included group, weekly average ambient temperature for the corresponding assessment period, and the interaction between group and temperature. Participant ID was included as a random effect. Ambient temperature was calculated as the 7-day average of the mean daily temperature in Tokyo during the corresponding assessment period. The daily mean ambient temperature in Tokyo during the study period is shown in Supplementary Figure S1. Because this analysis was exploratory, no multiplicity adjustment was applied. To aid interpretation of sample size adequacy, a post hoc power analysis was conducted using G*Power 3.1.9.7 for the primary outcome based on the per-protocol sample size. This analysis indicated that the per-protocol set provided >80% power (two-sided α = 0.05) to detect a between-group difference in Cohen’s d = 0.44, suggesting that smaller true effects may not have been reliably detected. The per-protocol set was prespecified as the primary population for efficacy evaluation because pre-excluding participants with protocol deviations or insufficient adherence that could compromise the integrity of the efficacy assessment was considered more appropriate for evaluating the efficacy of the test beverage. As a sensitivity analysis, the primary outcome was also evaluated using the full analysis set (FAS, *n* = 164), which approximates an intention-to-treat population. The FAS-based result for the primary outcome was consistent with the per-protocol analysis and is presented in Supplementary Table S1.

## 3. Results

### 3.1. Participant Analysis

**Figure 2:**
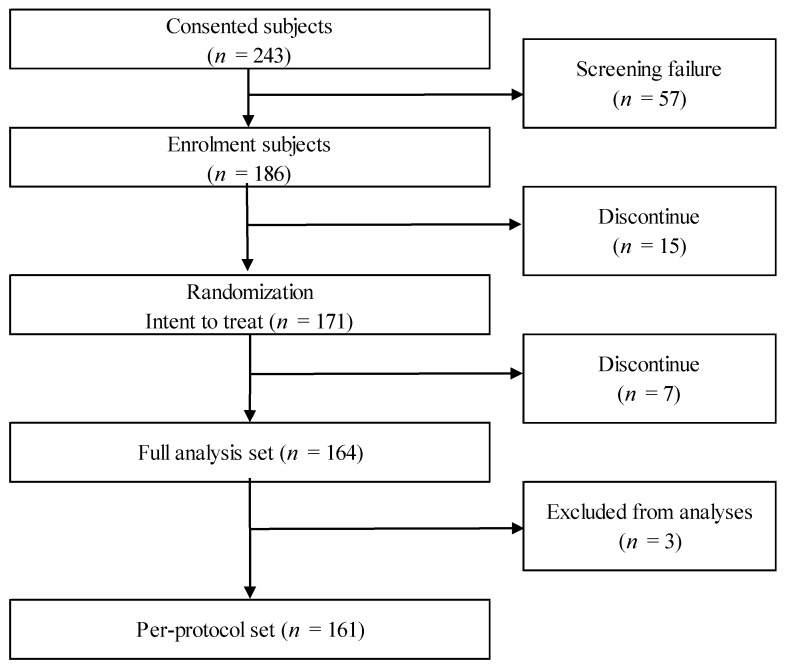
Study flow diagram.

A total of 171 healthy adults were enrolled in this study and comprised the intention-to-treat population. Of these, seven participants withdrew before starting the intake of the test foods. The remaining 164 participants who started the intervention were included in the full analysis set. Among the full analysis set, three participants were excluded from the efficacy analyses for predefined reasons: two participants showed poor compliance with test food intake (intake rate < 80%), and one participant had missing baseline data for the primary outcome assessed using the web-based daily log. Accordingly, the remaining 161 participants constituted the per-protocol set (Figure 2). All decisions regarding exclusion from the efficacy analysis population were made prior to unblinding and were finalized without knowledge of treatment allocation. The baseline characteristics of the participants are shown in Table 1.

### 3.2. Web-Based Daily Log

**Figure 3:**
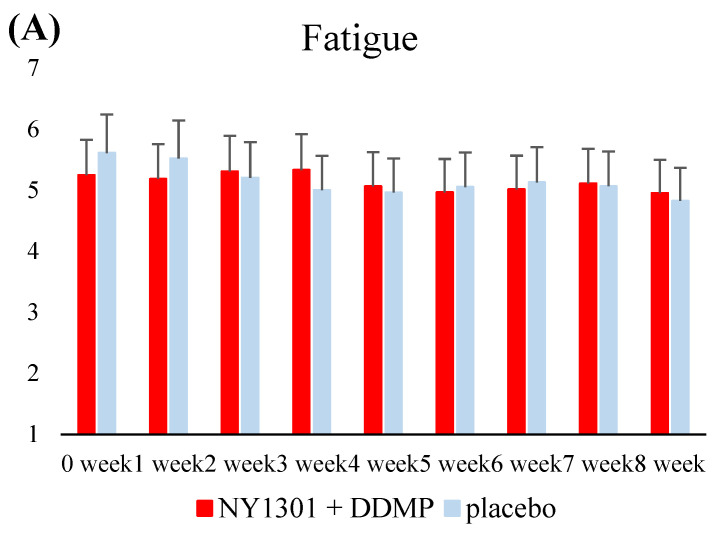

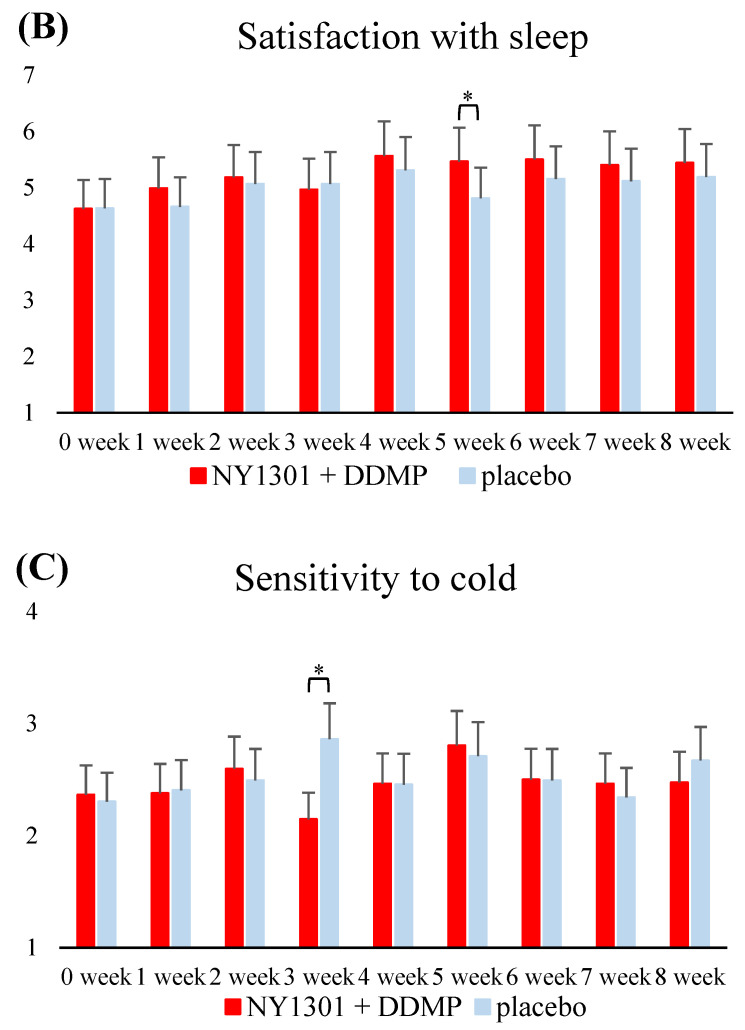
Effects of a beverage containing NY1301 and DDMP on web-based questionnaire results for (**A**) fatigue, (**B**) satisfaction with sleep, (**C**) and sensitivity to cold. Data are presented as means ± SEM. * *p* < 0.05 between groups determined by the Mann–Whitney U test.

Fatigue assessed using the web-based daily log, the primary outcome, is shown in Figure 3A. No significant between-group differences were observed at any time point. Satisfaction with sleep was significantly higher in the NY1301 + DDMP group than in the placebo group at week 5, and sensitivity to cold was significantly improved in the NY1301 + DDMP group compared with the placebo group at week 3 (Figure 3B,C). No significant between-group differences were observed in the other symptoms (dizziness/unsteadiness, tinnitus, stiff shoulders, lower back pain, appetite, irritability, general fatigue, motivation, and mental clarity).

### 3.3. Sleep Quality

**Figure 4:**
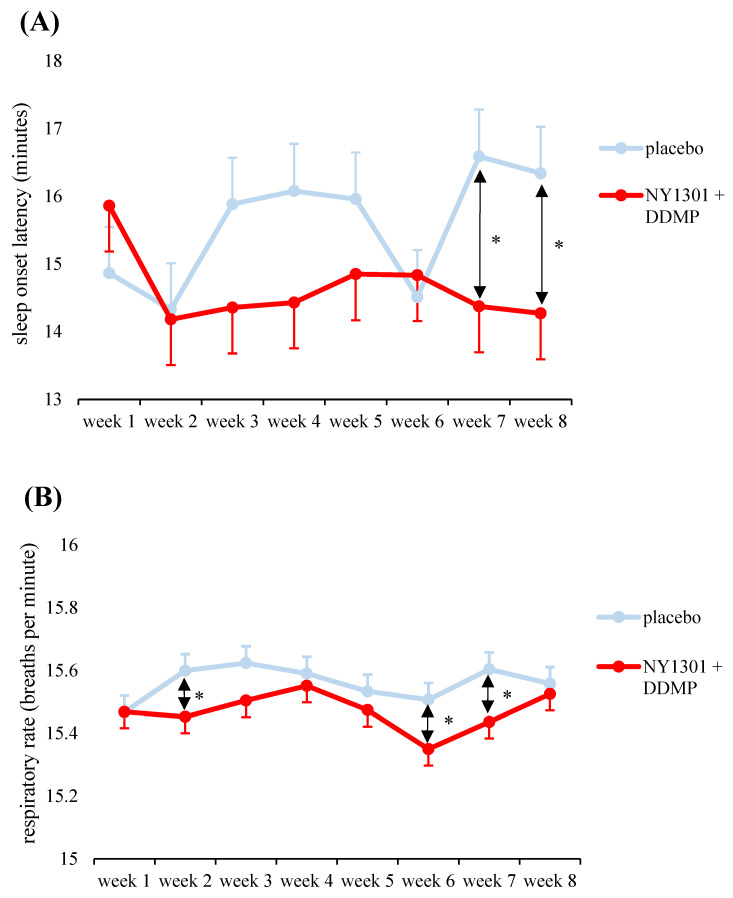
Sleep parameters in the NY1301 + DDMP and placebo groups during the study: (**A**) sleep onset latency, (**B**) respiratory rate. Data are presented as means ± SEM. * *p* < 0.05 between groups determined by linear mixed-effects model analyses.

In the NY1301 + DDMP group, sleep onset latency was significantly shorter than that in the placebo group at weeks 7 and 8 (Figure 4A). In addition, respiratory rate during sleep was significantly lower in the NY1301 + DDMP group than in the placebo group at weeks 2, 6, and 7 (Figure 4B). The daily mean ambient temperature in Tokyo during the study period is shown in Supplementary Figure S1. In an additional exploratory analysis, respiratory rate during sleep showed a significant group-by-temperature interaction (Supplementary Table S2). In the placebo group, respiratory rate during sleep increased as the weekly average ambient temperature rose, whereas this temperature-associated increase was attenuated in the NY1301 + DDMP group.

### 3.4. Questionnaire

**Figure 5:**
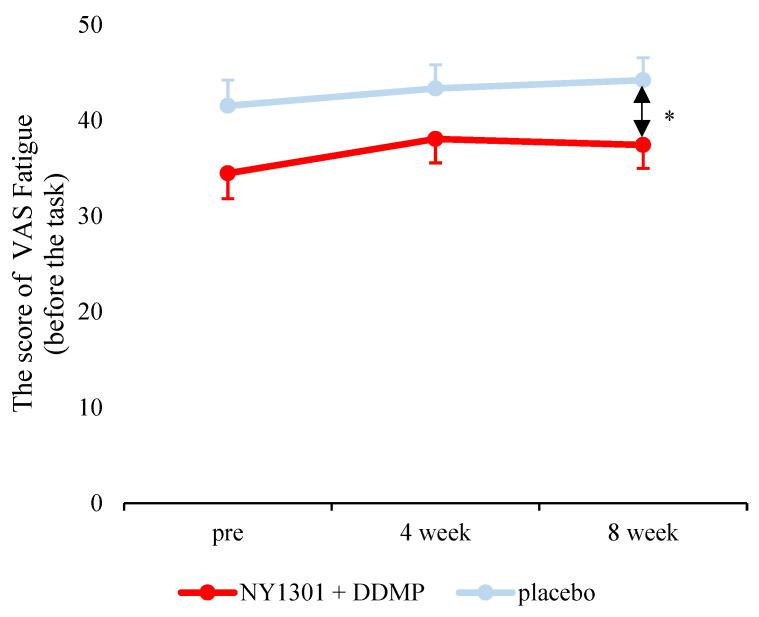
Effects of a beverage containing NY1301 and DDMP on the visual analogue scale (VAS) scores for fatigue before starting the calculation task. Data are presented as means ± SEM. * *p* < 0.05 between groups determined by the Mann–Whitney U test.

At the 8-week measurement, the NY1301 + DDMP group showed significantly lower VAS scores for fatigue before starting the calculation task compared with those in the placebo group (Figure 5). No significant between-group differences were observed in POMS2 and IPAQ short-form scores.

### 3.5. Calculation Task

There were no significant differences in performance metrics (total number of responses, number of correct responses, accuracy rate and performance score which was defined as number of correct responses × accuracy rate) for the calculation task.

### 3.6. Stress Biomarkers

While the NY1301 + DDMP group showed significantly higher salivary α-amylase activity at 10 min post-task during the pre-measurement, no significant differences were observed between groups at the 4-week and 8-week measurements. No significant differences were found in salivary cortisol levels throughout the study period.

### 3.7. Autonomic Nervous System Function

**Figure 6:**
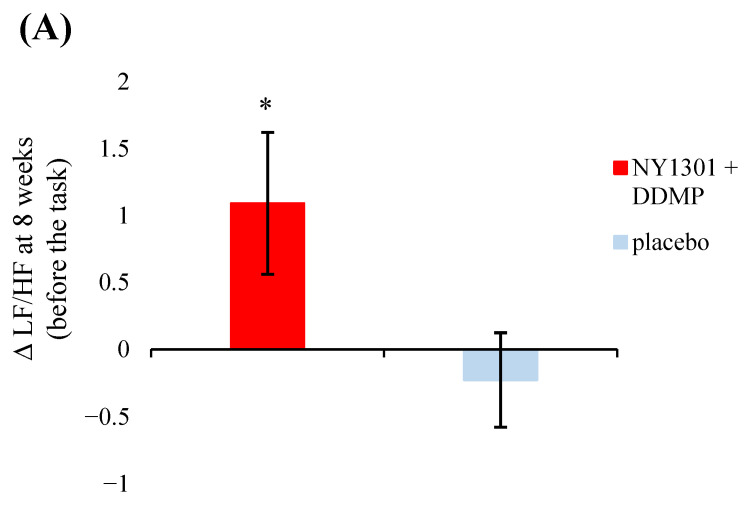

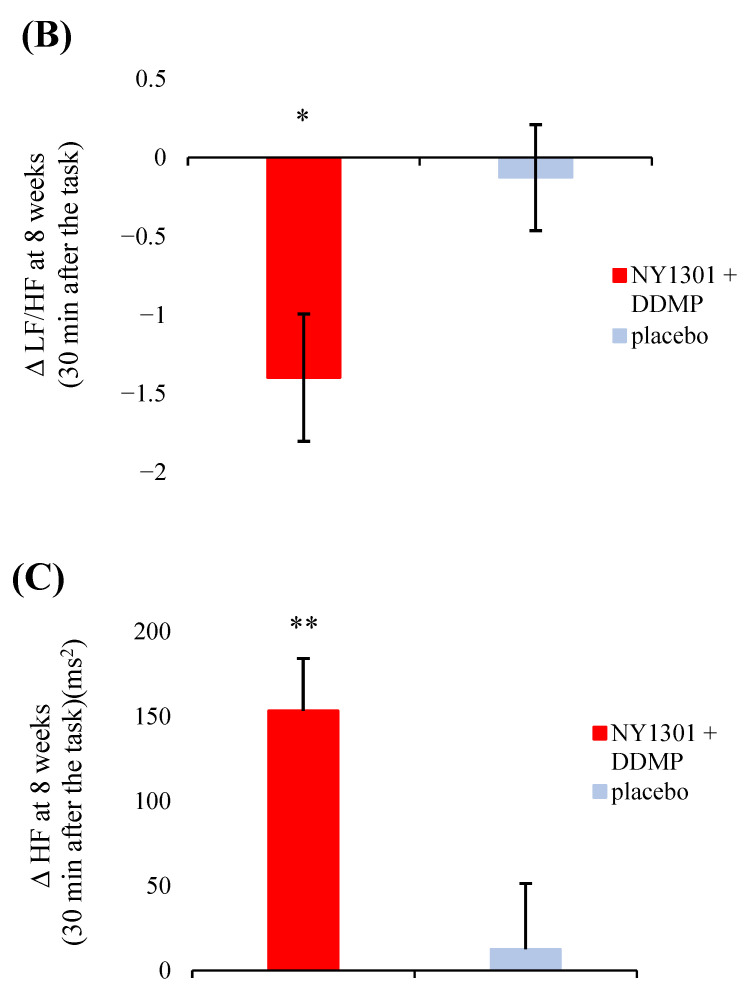
Changes in autonomic nervous system function: (**A**) changes in LF/HF from pre-task to immediately after the task, (**B**) changes in LF/HF from immediately after the task to 30 min post-task, (**C**) changes in HF from immediately after the task to 30 min post-task. Data are presented as means ± SEM. * *p* < 0.05, ** *p* < 0.01 between groups determined by Welch’s *t*-test.

At the 8-week measurement, the autonomic nervous system response to the calculation task differed significantly between the groups. Regarding the LF/HF ratio, the increase from pre-task to immediately after the task was significantly greater in the NY1301 + DDMP group than in the placebo group (Figure 6A). The decrease in the LF/HF ratio from immediately after the task to 30 min post-task was also significantly greater in the NY1301 + DDMP group (Figure 6B). Furthermore, for the HF component, the NY1301 + DDMP group showed a significantly greater increase from immediately after the task to 30 min post-task compared with that in the placebo group (Figure 6C).

### 3.8. Test Food Safety

No serious adverse events occurred during the study. All adverse events were judged by the clinical investigator to be unrelated to the study products.

## 4. Discussion

We investigated the effects of the combined intake of *L. paracasei* subsp. *paracasei* NY1301 and DDMP on summer heat fatigue-related symptoms in healthy adults. No significant between-group difference was observed in the prespecified primary outcome, subjective fatigue assessed by a web-based daily log. However, favorable between-group differences were observed in several secondary outcomes, including lower pre-task VAS fatigue at week 8, improved sleep-related parameters, and greater autonomic changes during stress recovery. These secondary findings should be interpreted cautiously. Notably, the consistency of these favorable differences across independent measurement domains—subjective fatigue under standardized conditions, objective sleep parameters, nocturnal respiratory rate in relation to ambient temperature, and post-stress autonomic recovery—supports their physiological plausibility, while their clinical relevance requires confirmation in independent studies.

The difference in results between the web-based daily log and VAS analyses may be explained by the difference in measurement conditions. The web-based daily log was recorded repeatedly during the participants’ daily lives and may therefore have been affected by daily events, such as workload, sleep duration, and psychological stress [28,29]. The VAS was assessed at the clinic under standardized conditions. Therefore, the effect of the test beverage on subjective fatigue may have been detected more clearly in the clinic-based assessment than in the daily log.

The improvement in sleep-related parameters is an important finding of this study. Summer heat is known to impair sleep quality [30,31], and poor sleep is associated with greater fatigue under hot conditions [3,32]. In the present study, the NY1301 + DDMP group showed improvements not only in sleep satisfaction in the web-based questionnaire but also in objective sleep-related indices, such as sleep onset latency and respiratory rate during sleep. These results suggest that the combined intake of DDMP and NY1301 contributed to improvements in both subjective and objective aspects of sleep during the summer season. The between-group difference in sleep onset latency was modest. However, the participants were healthy adults whose mean sleep onset latency was already within the range generally considered normal for good sleep quality (i.e., within 30 min) [33]. The observed reduction corresponded to an improvement of approximately 11–13% from baseline. The previous study reported a comparable reduction in sleep onset latency together with improvements in next-morning sleepiness and mood [34]. In the present study, the significant reduction in pre-task fatigue VAS at week 8 is consistent with this interpretation. Nonetheless, the absolute between-group difference of approximately 2 min in sleep onset latency is small, and the minimal clinically important difference for this measure in healthy adults has not been firmly established. The clinical relevance of this finding therefore remains uncertain and warrants confirmation in independent studies. Previous validation studies comparing the Oura Ring with polysomnography have shown reasonable agreement for sleep onset latency at the group level [35,36]. The Oura Ring has also been used in recent clinical trials to evaluate sleep-related outcomes [37,38]. Therefore, the sleep findings derived from the Oura Ring in the present study provide supportive objective information regarding the effects of DDMP and NY1301 on sleep.

The autonomic nervous system findings further support this interpretation. At week 8, the NY1301 + DDMP group showed a significantly greater increase in the LF/HF ratio from pre-task to immediately after the mental arithmetic task, followed by a significantly greater decrease during the recovery period. In addition, the NY1301 + DDMP group showed a significantly greater increase in HF from immediately after the task to 30 min post-task. Mental arithmetic is known to induce cardiovascular and autonomic responses as a psychological stressor, including changes in HRV components, such as an increase in LF/HF during task engagement [39]. In motivated cognitive performance tasks, adequate physiological mobilization to meet task demands has been linked to greater task engagement and more favorable performance [40], whereas blunted sympathetic activity has been associated with poorer cognitive performance and greater behavioral disengagement [41]. HF is related to parasympathetic activity [42]; accordingly, our results suggest that parasympathetic activity increased more markedly during the post-task recovery period in the NY1301 + DDMP group. Recovery from psychological stress has been associated with increased vagal modulation [43]. Taken together, these findings may indicate greater autonomic reactivity during task exposure and greater parasympathetic rebound during recovery in the NY1301 + DDMP group. However, these results should be interpreted as exploratory evidence of altered autonomic recovery rather than definitive proof of mechanism. The magnitude of the observed autonomic shifts was comparable to that reported in previous experimental stress and relaxation paradigms [39,44], suggesting that these findings were physiologically meaningful rather than trivial. Nevertheless, the LF/HF ratio is influenced by both sympathetic and parasympathetic activity and does not provide a direct measure of sympathovagal balance; accordingly, the present LF/HF findings should be interpreted cautiously, and the clinical relevance of the observed differences in healthy adults remains uncertain and requires confirmation in future studies.

We also detected an improvement in sensitivity to cold and observed a temperature-related difference in respiratory rate during sleep. Sensitivity to cold may be partly related to peripheral circulatory or thermoregulatory responses [45,46]. Respiratory rate is a physiological variable that is sensitive to heat, physical effort, and fatigue, and is used as an indicator of physiological strain in both healthcare and sport settings [47]. In addition, nocturnal respiratory rate is considered an objective physiological parameter reflecting physiological state during sleep [48], and elevated values may indicate greater residual physiological burden during the night [49]. In the present study, respiratory rate during sleep showed a significant group-by-temperature interaction: it increased with rising ambient temperature in the placebo group, whereas this temperature-associated increase was suppressed in the NY1301 + DDMP group. These findings suggest that the participants were exposed to summer environment-related physiological burden during the study period and that this burden may have been attenuated in the NY1301 + DDMP group. Taken together with the HRV findings, these results may be consistent with an effect of the combined intake of DDMP and NY1301 on autonomic regulation under both task-induced and daily physiological conditions, although the between-group difference in respiratory rate during sleep was small in absolute terms (approximately 0.2–0.4 breaths/min) and its clinical relevance in healthy adults remains uncertain. In this context, summer heat fatigue should be understood not as an acute heat illness such as heatstroke, but rather as a state of nonspecific complaints occurring during the summer season [5,6]. The improvements observed in pre-task fatigue VAS and sleep onset latency are therefore reasonably interpreted as improvements in symptoms related to summer heat fatigue under summer conditions, rather than merely nonspecific changes unrelated to the seasonal environment.

Summer heat fatigue is considered to be associated with autonomic nervous system dysregulation [50,51]. Under heat stress, sympathetic predominance may contribute to fatigue and poor sleep quality [40,50,51]. Passive heat exposure has been shown to increase cardiovascular strain and impair executive function [52], potentially because of the cumulative effects of autonomic dysregulation and increased thermal discomfort. Previous studies have further shown that heat stress induces oxidative stress and impairs intestinal function, both of which may contribute to autonomic dysregulation [8,9,10,11,12]. An important feature of this study is the focus on a combination of two food-derived components with potentially complementary mechanisms of action. NY1301 is a probiotic strain reported to improve bowel function [21], and DDMP is an antioxidant compound identified in LAB-fermented beverages [17]. Taken together, these observations suggest that NY1301 and DDMP may support autonomic nervous system regulation through distinct but complementary mechanisms, i.e., DDMP may target oxidative stress-related pathways, whereas NY1301 may support gut-related homeostasis. This combined probiotic-antioxidant approach represents a novel nutritional strategy for summer heat fatigue-related symptoms. Because oxidative stress markers and gut microbiota-related parameters were not directly measured in this study, the proposed mechanisms involving oxidative stress reduction and gut-related homeostasis remain hypothetical and should be verified in future studies.

All adverse events reported during the study period were judged by the clinical investigator to be mild and unrelated to the study products, supporting the safety of combined NY1301 and DDMP intake for 8 weeks.

Our study had several limitations. First, no statistically significant between-group difference was observed in the primary outcome, subjective fatigue assessed using the web-based daily log. Therefore, the anti-fatigue effect of the test beverage should be interpreted with caution. Second, no formal multiplicity adjustment was applied to the analyses of multiple secondary outcomes. Accordingly, the possibility of false-positive findings cannot be excluded, and these secondary findings should be interpreted as exploratory. Third, the sleep parameters measured using the Oura Ring were limited, and more detailed evaluations using EEG are needed in future studies. Fourth, because the study did not include a DDMP-alone or NY1301-alone group, the contribution of each component could not be determined. Future research is warranted to disentangle the individual effects of DDMP and NY1301 and to determine whether their combination provides additive or synergistic benefits. Fifth, because this study included only healthy Japanese adults, the generalizability of the findings to other populations remains limited. Sixth, although measures to minimize bias were implemented, the potential influence of industry sponsorship and sponsor-affiliated authors cannot be completely excluded and should be considered when interpreting the findings. Independent replication studies conducted by researchers without financial ties to the sponsoring companies are warranted to further strengthen the robustness and external validity of the present findings.

## 5. Conclusions

The primary outcome, subjective fatigue assessed using the web-based daily log, was not significantly different between groups during the summer study period. However, combined intake of NY1301 and DDMP was associated with favorable changes in selected sleep-related outcomes and post-stress autonomic recovery parameters. Further studies are required to clarify the effects on fatigue and to determine the clinical relevance of the sleep- and autonomic-related findings. Taken together, these findings provide a preliminary physiological rationale for the combined intake of NY1301 and DDMP as a nutritional approach to summer-related physiological burden in healthy adults and warrant confirmation by independent trials incorporating mechanistic biomarkers and factorial component designs.

## Figures and Tables

**Table 1:** Baseline characteristics of participants.

	FAS		PPS	
	NY1301 + DDMP (*n* = 82)	Placebo (*n* = 82)	NY1301 + DDMP (*n* = 82)	Placebo (*n* = 79)
Male	*n* = 38	*n* = 39	*n* = 38	*n* = 38
Female	*n* = 44	*n* = 43	*n* = 44	*n* = 41
Age (years)	40.8 ± 6.0	40.7 ± 6.2	40.8 ± 6.0	40.7 ± 6.3
BMI (kg/m^2^)	22.9 ± 2.8	22.9 ± 2.6	22.9 ± 2.8	23.0 ± 2.6

Data are shown as means ± SD. *n* is the number of participants. NY1301, *Lacticaseibacillus paracasei* subsp. *paracasei* NY1301; DDMP, 2,3-dihydro-3,5-dihydroxy-6-methyl-4H-pyran-4-one; BMI, Body mass index. FAS, Full analysis set; PPS, Per-protocol set.

## Data Availability

The study protocol is publicly available through the UMIN Clinical Trials Registry (UMIN000057607). The anonymized individual-participant dataset will be deposited in a public data repository upon publication and is also available from the corresponding author on reasonable request.

## Supplementary Materials

The following supporting information can be downloaded at https://www.mdpi.com/article/10.3390/jcm15114175/s1. Supplementary Figure S1. Daily temperature trends in Tokyo during the intervention period (July to September 2025). Supplementary Table S1. Time-course results of the primary outcome (subjective fatigue assessed using the web-based daily log) in the full analysis set (FAS, n = 164). Supplementary Table S2. Results of the linear mixed-effects model for respiratory rate during sleep.

## Abbreviations

DDMP	2,3-dihydro-3,5-dihydroxy-6-methyl-4H-pyran-4-one
LAB	lactic acid bacteria
IPAQ	International Physical Activity Questionnaire
POMS2	Profile of Mood States 2nd Edition
VAS	visual analog scale
