# Supplementary material for: Combined NY1301 and DDMP on Sleep and Autonomic Function in Summer: A Randomized Clinical Trial
Source: J Clin Med. 2026 May 28;15(11):4175. doi: 10.3390/jcm15114175 (PMC13257763; doi:10.3390/jcm15114175)
Supplement: Supplementary file 1 [file jcm-15-04175-s001.zip › jcm-4266637-supplementary.pdf]

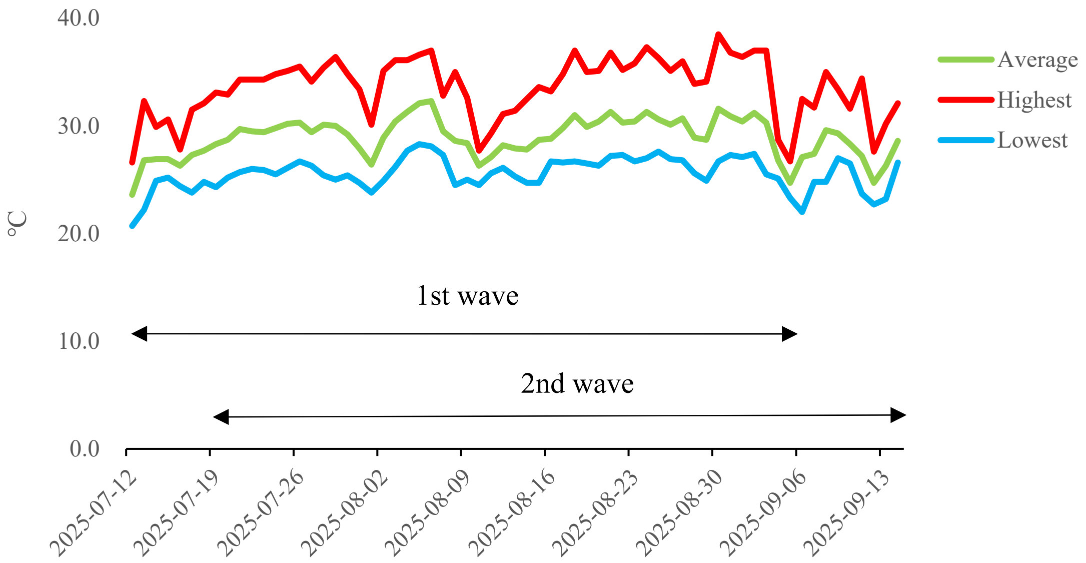

Supplementary Figure S1. Daily temperature trends in Tokyo during the intervention period (July to September 2025). The lines indicate daily mean, highest, and lowest ambient temperatures, respectively. Shaded segments labeled "1st wave" and "2nd wave" indicate the respective intervention periods for the two separate participant cohorts during the study. Data were retrieved from the official database of the Japan Meteorological Agency (https://www.jma.go.jp/).

Outcome	group	week 0	week 1	week 2	week 3	week 4	week 5	week 6	week 7	week 8
fatigue	NY1301 + DDMP	5.24 ± 0.22	5.18 ± 0.20	5.30 ± 0.22	5.33 ± 0.22	5.06 ± 0.22	4.96 ± 0.23	5.01 ± 0.19	5.11 ± 0.20	4.95 ± 0.21
	placebo	5.62 ± 0.19	5.52 ± 0.18	5.22 ± 0.18	5.01 ± 0.20	4.99 ± 0.19	5.06 ± 0.19	5.12 ± 0.22	5.10 ± 0.23	4.88 ± 0.21
	p . value	0.272	0.26	0.624	0.219	0.737	0.816	0.748	0.909	0.632

Supplementary Table S1. Time-course results of the primary outcome (subjective fatigue assessed using the web-based daily log) in the full analysis set (FAS, $n = 164$). Data are presented as means ± SEM. *p*-values were determined by the Mann–Whitney U test for comparison between the NY1301 + DDMP group and the placebo group at each time point.

Outcome	item	β	βstd	SE	95%CI	F value	*p* value
respiratory rate	group	-0.022	-0.014	0.121	[-0.260, 0.217]	0.032	0.859
	temperature	0.016	0.012	0.009	[-0.001, 0.033]	3.442	0.064
	group × temperature	0.021	0.016	0.009	[0.004, 0.038]	5.722	0.017

Supplementary Table S2. Results of the linear mixed-effects model for respiratory rate during sleep. The dependent variable was respiratory rate during sleep measured by the wearable device. Fixed effects included group, weekly average ambient temperature, and the group × temperature interaction; participant ID was included as a random effect. Group-related coefficients were coded as deviations of the placebo group from the grand mean; therefore, a positive group × temperature coefficient indicates that the temperature-related slope was greater in the placebo group than the overall average slope, and correspondingly smaller in the NY1301 + DDMP group. β, unstandardized regression coefficient; βstd, standardized regression coefficient; SE, standard error; CI, confidence interval.
